# The Fundamental Part of Anatomy—The Bones

**Published:** 1881-07

**Authors:** J. L. Cilley

**Affiliations:** Demonstrator of Anatomy


### THE FUNDAMENTAL PART OF ANATOMY—THE BONES.

(Anglo-Saxon, Ban).

*A Lecture delivered in the Spring Course of the Medical College of Ohio, Session of* 1881.

By J. L. Cilley, M. D., Demonstrator of Anatomy.

The hard and dry 200 bones, which compose the adult skeleton, are always " full of meat and juicy," for the ardent and attentive observer, engaged in the study of anatomy. They furnish much food for reflection. The bones live for the physiologist, for the pathologist, and most essentially for the surgeon. Ezekiel, the prophet, was told to say " O, ye dry bones, ye shall live."

The word skeleton expresses only a physical condition, and applies merely to the bones, when used, as referred to now, in a limited sense. It comes from the Greek word σκελλω (I dry).

The fate of many beginners in the study of medicine is such, that at the very threshold, they acquire a distaste for this fundamental part of anatomy, and the bones acquire a bad name, so that any excuse is thought good enough for throwing them aside. Through the carelessness and incompetency of would-be preceptors, possessed of

" Skulls that cannot teach and will not learn"

the study of this important branch of medicine becomes irksome, and the hours devoted to it dreary and tedious. The day of beginning the study of medicine is lamented as "dies infaustus."

A very interesting study is found in the terms and expressions used to describe the bones independently of their physiological, pathological and surgical bearings. Many of them are very appropriate in their application, but tradition and fancy has had much to do with some of them, so that their etymology is of practical interest. As it is somewhat connected with our subject, we will refer to an idea, prevalent among the laity, viz.: that the number of the ribs is *not the same* in the two sexes. Students are often known to look for a confirmation of this belief, in the dissecting-rooms, but it has *not* yet been found. There is no anatomical basis for the belief.

In all systematic works on osteology, the description of the "spine" or "vertebral column" comes first. These terms are expressive and good. Most certainly is it a column, a structure of support, and were it straight, instead of curved and elastic as it is, it would yield under a weight, only *one-ninth* of what it now sustains. It is vertebral, from the Latin *verto* (I bend-turn); for, in its joints below the dentata, there is traced the double ball and socket type of articulation.

Its spinous feature will not disappoint him who looks for the application of the term spine, from *spina* (a thorn). Its more than 170 processes, and many of them bifurcated, show that he who first so termed it, was justified in doing so.

The function and structure of this portion of the skeleton readily suggested these terms; but mythology, tradition and fancy, aided in designating the so-called atlas, sacrum and coccyx. The origin of the latter term, from the greek κύκκυξ (cuckoo), is hardly justifiable from any resemblance to the bill of the bird. Huckle, or knuckle-bone is better, and *whistle-bone* by some is somewhat suggestive, because of its locality at the lowest part of the column.

In mythology, Atlas supports the vault of heaven; hence the application of the term to the first bone of the column, which supports the head. Some of the older anatomists, however, termed the last cervical, the atlas.

An expression, meaning sacred, as applied to the largest bone of the vertebral series, antedates the time of Hippocrates in its use by the Greeks. The Latin word sacrum is the equivalent of the Greek ἱερόν (connected with the gods — sacrifices, etc.), and tradition tells us that the ancients sacrificed the lower part of the loins, and that the bone here referred to, was burnt as a votive offering.

It has been intimated on good authority, that the priests, who officiated at the altars, would share with the gods, in what was sacrificed; and that this bone, together with what was attached thereto, was sacred to their appetites. (Hyrtl). This is the largest bone in the column, and the fact that the ancients so regarded large things, may have had something to do with the adoption of the term sacred; also that the lower portion of the spine was held in veneration among the Arabians, and that, among the Jews, superstition held that the sacrum had an incorruptible nature and holy function after death. It is through tradition and superstition then, that a sacred character has been attached to the "sacrum;" but there are good reasons for believing that ἱερὸν the Latin equivalent for which is sacrum, is a corruption of the Hebrew word Heron, the signification of which is conception, gestation, and the process of parturition. The relation which this bone bears to these processes is readily seen.

This wonderful *central part* of the axial skeleton is a marvellous column, giving attachment as it does to sixty-two pairs of muscles and supporting not only the weight of the body, but of all that can be carried on the head and back and in the upper limbs. Its structure and function are well expressed in the terms used in connection with it, and it is very interesting to note how much mythology, fancy, tradition and superstition, has had to do with the origin of terms used in connection with several of its parts.

The bones of the cranium and face come next in order. Of the expressions cranium and face, what is to be observed?

The Cranium.—The eight bones which compose it, form, by the way they are united, a complete helmet for the necessary protection of the brain. It might be said, and very plausibly, that cranium has its origin in κράνος (a helmet); but in the old Greek word καρ (the hair of the head), we have the probable root of κρᾶνὶον (the skull). Is it not fair to presume that in the hairy feature of the head, there is found the most probable etymology of the cranium?

Among the eight bones we have the frontal (front) or forehead bone, which in by-gone literature was called *os coronale* (crown), *os invere-cundum*; (immcdest), and *os sensus communis.* We might revive this last term by reason of this bone's relations to the anterior part of the brain. A naval term has been applied, viz. : puppies (poopdeck or stern), and in this connection we may state that the word meaning prow, has been applied to the occipital bone. The application of stern and prow to these two bones, respectively, is good. The stern, was the highest part of the deck, and the place where the pilot or *gubernator* had his outlook, and it is well known how important the prow was in ancient maratime affairs; especially in naval conflicts. The term bregmatic, from Βρέγμα (a fountain), has been used in connection with the frontal and parietal bones. An explanation of this lies in the fact that these three bones form the boundaries of the anterior fontanelle, which the ancients imagined served to evacuate the superfluous moisture of the brain.

It is quite apparent that the parietals serve as protecting walls to the encephalon. Hence the word signifying a wall.

In the expression ossa-temporalia (time bones), reference is had to the hair first becoming gray on the temples; but some of the older writers recognized the fact, that this was no sign of age, and therefore called the temporals "ossa mendosa."

The ethmoid gets its name from the great number of holes in the cirbriform lamella, but it is such a spongy bone, that spongiform, which has been sometimes used, would be more appropriate.

The sphenoid is certainly wedged in at the base of the skull. Its figure is so irregular that multiform would be better. Its resemblance to a bat with wings extended, is very faint. Finally we have the occipital, essentially *the* bone of the head. Its name (a compound of *ob* and *caput*) indicates this. It bears the whole weight of the head, and is in relation with nerve centres most essential to life. Disease very rarely attacks it, but in rickety children it undergoes softening and other alterations.

THE FACE.—This word comes from the latin *facies* (face, countenance), which has its origin in *facio* (I make, act, form). It is a very suggestive word in its application to this part of the body. One meaning of the word *facies*, is to show a thing in its true light. This is good when it is remembered that the face is the mirror of the soul, and so often the key in the determination of character.

The older writers divided the face into the upper and lower jaws—maxillæ,—and Celsus applied "maxilla" to the lower jaw and gave to the upper the name of *mala.* Maxilla, however, is the diminutive of *mala* (cheek-bone, jaw), and the latter is derived from *mando* (I chew, I grind with the teeth). *Mala* has also been rendered teeth. The function of these parts makes such terms very appropriate. Nowadays *mala* designates the cheek boone; and upper and lower maxillæ, the teeth-bearing portions of the face.

Among the irregular mass of bones which compose the face, we find such as the nasal (nose) and the turbinated—from *turbinatus* (cone-like), and lachrymal or os unguis. In figure and magnitude the latter resembles the finger-nail, and has the tears passing upon it. Hence the terms.

The resemblance of the vomer to a plowshare, is not altogether fanciful. The word for palate, *palatum,* is probably an original word, but may possibly be connected in its origin with *palor* (I wander up and down). Recall the mechanism of deglutition, and think how much normally passes down against this bone. A word with such a meaning seems very appropriate, since an adult ingests more than a ton of food annually.

There is a little bone off by itself, in the region of the throat, bearing a resemblance to the Greek letter *υ*, hence hyoid, and known by some as the bone of Adam's apple.

The thorax or chest. Both of these terms are of Greek origin. Our word, chest, Latin *cista,* comes from the Greek *κίστη* (a box), and we should not laugh when the Irish speak of the chist. From its derivation, both Latin and Greek, it is certainly more classical.

In ancient armor a breast-plate covered the whole forepart of the trunk. The Greek word for cuirass is *θώραξ* (a breast-plate). Sufficient protection for the heart and lungs is imperative, and the term *costæ,* from *custodire* (to defend, protect), is used to designate the ribs—and solid, in contrast with the cartilages and muscles, in which it is imbedded, do we find the sternum from στέρνον (solid).

The Pelvis.—The resemblance of this part of the skeleton to an ancient basin, justifies the use of the word pelvis, from πέλις; but all efforts failed of a name for its main bone. Innominate it has been and innominate it is; but a part of it had to do with the flank, hence *ilia* (flank); while another portion having a supporting duty to perform when we sit, suggested ischium. The third anterior portion is known as the os pubis, and also as the os pectini. We fail to recognize its resemblance to a comb, which *pecten* means; but Celsus used this expression, copying from Juvenal, who uses the word pecten in connection with "the hair about the privy parts." That portion of the body with which this portion of the bone is in relation, is generally bedecked with hair at the time of puberty. Hence is derived the designation os pubis, from the Latin *pubes* (the down or soft hair which grows on young people when they come to the age of puberty). The corresponding word in Greek is ἥβη, one meaning of which is "the outward signs of manhood."

In the upper extremity, there is the so-called clavicle, from the Latin *clavis* (a key). The resemblance to an ancient key of some form or other is rather ambiguous, but in its relations to the arch of the shoulders, across the chest, the above certainly is the key of the situation, in the support which it gives to the shoulders. All know that the shoulder falls when this bone is broken.

Scapula. There is a Latin word *scoptula*, which means shoulder bones. Scapula is probably derived from it, and how easy to get *scutulum* (a little shield) from *scoptula*, since the bone is more or less a shield. Scutulum opertum it has been called.

What is our word arm? There is a word *armus* (the shoulder; used most commonly of brutes, though sometimes of man) and the Greek word ἀρμὸς, which refers to the shoulder limb. The first three letters of either of these words furnish arm. How simple and plain is its derivation, and how few stop to consider it.

The word humerus is of Greek origin, from the two Greek words ὦμος (shoulder) μηρός (the ham). How evidently is this the os humeri, the bone of the shoulder ham, compounded of ω and μηρὸς.

In the forearm we have the radius and ulna. The radius closely resembles a rod or spoke, and in the Greek word ῥάβδός, which means a stick, etc., we doubtless have the derivation of radius. The application is good. Several words, the literal meaning of which is elbow, have been used to designate the ulna. They are as follows: ulna ὠλένη, *cubitum*, and μίβῖτον. The radius does not enter into the formation of the elbow-joint, hence the ulna is more properly the elbow bone. If we measure the distance from the elbow to the tip of the middle finger, we shall find the distance to be just about eighteen inches—a cubit's length—hence it is called the cubit bone. In the word carpus, we have the wrist, said to be derived from a word meaning to connect together, the hand, which is the seizing or grasping part—with the forearm. The meta-carpals have their proximal ends among, in, between, after or next to the carpals. The prefix μετὰ signifies all these.

As to the phalangeal bones, it is readily understood why they are so called, both in the hand and in the foot. In the formation of a phalanx (from φάλαγξ) men are arranged several lines deep —so are these bones, and in ancient times this word φάλαγξ had for one of its meanings the bone between two of the joints of the fingers and toes. The Latin word *internodium* (*inter* and *nodus*), the space between two joints, has been used in connection with these bones—but more especially with the divisions of the thumb.

THE LOWER EXTREMITY.—Our word thigh comes from the word *thic* (thick), and its Latin equivalent is *femur*, which means the thigh. It is incorrect to call the thigh-bone anything else than os femoris.

In the knee-joint we find a little bone called patella—diminutive of the Latin *patera* (a broad, shallow cup or bowl). Being something like a disc, it has been called disciforme. Celsus termed it μύλη (a mill), possibly because of its resemblance to a millstone. It has also been termed *oculus genu*. In function it is a sesamoid bone, found as it is in the tendon of the quadriceps extensor, but its size for bids its being linkened to the small seed of the sesame, an Eastern leguminous plant. More than 150 years ago, it was known as the *rotula*—diminutive from *rota* (a little wheel). Its method of gliding in the various movements of the knee-joint, is by no means simple. In the leg we have what is known as the shin-bone. Shin is of Anglo-Saxon origin, coming from *scina*, and instead of shin, it could more properly be called skin-bone. It is poorly protected, covered by skin only, and therefore easily injured.

In the etymology of its Latin name, *tibia* (a pipe), we get a little history of our musical instrument—the flute. The first straight musical instruments with holes were probably made of bone. And it is readily seen how easy it would be to make a clarionet out of a human tibia or shinbone. The tibia was the commonest musical instrument of the Greeks and Romans. Players on the tibia were called *tibicines* (from *tibia* and *cano*); and just here the thought occurs, could we call foot-ball players who take such pleasure in kicking each other's shins, tibicines, and the performance a tibicinium, because of the painful, lugubrious sounds which are heard so often on such occasions?

In the relations of the fibula to the tibia, it is readily seen how like a clasp of a brooch or brace it is. Both of which terms are embraced in the word *fibula.* The origin of this word is possibly from *figo,* I fix. We also have the expression, peroneal. This is from the Greek word περόνη (anything pointed for piercing or pinning), which comes from πείρω (I run through, pierce).

Generally speaking, the Greek word ταρσός means the foot. Another meaning is, any broad, flat surface. Ταρσός ποδός means the flat of the foot, the part between the toes and heel, including both the tarsal and meta-tarsal bones. Of the metatarsal and phalangeal bones the same may be said as was said of the corresponding bones of the hand.

Seven bones compose the tarsal, two of which are very large, viz.: the astragalus and os calcis. The former is known as the die bone, because it has been said that the ankle-bones of inferior animals were used as dice before they were made of other materials. The Latin word for the same bone is *os tali.* The Greek word αστράγάλος and the Latin *os tali* both mean the ankle. The largest bone of the foot is the os calcis. *Calx* is the Latin for heel—an entirely different word from calx of Greek origin, which means lime or chalk. *Calcar pedis* (the spur of the foot) has been applied to the heel bone.

The remaining five bones are composed of the boat-like (scaphoid) bone, three wedge-like (cuneiform), and the cube-like bone—the cuboid.

We must not fail to call your attention to the eight bones which compose the carpus. These are arranged in two rows more or less parallel, with four in each row. We find here also a scaphoid, from *σκάφη* (a skiff), and a cuneiform (from the Latin *cuneus* a wedge). The largest of all is known as the os magnum. And by reason of a hook-like process on another, there is seen the unciform (from *uncus*, a hook, and *forma*, form). The trapezium (from *τράπεζα*, a table) is there, and the bone something like it, the trapezoid. A crescent like bone, the semi-lunar is seen among them, and finally that affair, a mere sesamoid bone, which, from its resemblance to a pea, is known as the pisiform, from the Latin *pisum*, a pea.

We had almost omitted the *œsicula auditus*—the little bones of the ear. They must not be passed by. A glance shows that they closely resemble a stirrup, an anvil, and a hammer respectively. Our ancestors named them *stapes*, *incus*, and *malleus*. From their shape they could not be more appropriately named. And he who is familiar with Latin would, before ever seeing them, infer from their names a resemblance on their part to a stirrup by one, and an anvil and hammer by each of the other two.

In conclusion it is thus seen what a field for study the etymologist has in connection with so many of these terms to which your attention has been directed. The origin and derivation of anatomical expressions furnish a wide range for thought. You perceive how many of them are of Greek and Latin origin, and cannot help realizing that he who knows the so-called "dead" languages has a great advantage over him who never studied them. It is a matter of wonder and surprise that they who are unversed in the classics, get along as well as they do in medicine. From this review, I trust you will be impressed with the necessity of the study of Latin and Greek. Make it a part of your studies, for a knowledge of even their rudiments will aid you considerably.

